# Prevalence of breast-related symptoms, health care seeking behaviour and diagnostic needs among women in Burkina Faso

**DOI:** 10.1186/s12889-018-5360-6

**Published:** 2018-04-04

**Authors:** Leonie Ströbele, Eva Johanna Kantelhardt, Timongo Francoise Danielle Traoré Millogo, Maurice Sarigda, Jürgen Wacker, Kirstin Grosse Frie

**Affiliations:** 1 Martin-Luther-University Halle-Wittenberg, Institute for Medical Epidemiology, Biostatistics and Informatics, Magdeburger Straße 8, 06112 Halle, Germany; 2 Department of Gynaecology, Martin-Luther-University Halle-Wittenberg, Ernst-Grube-Str. 40, 06097 Halle, Germany; 3 Unité de Formation en Sciences de la Santé, Université Ouaga 1 Professeur Joseph Ki Zerbo 03 B.P. 7021, Ouagadougou, 03 Burkina Faso; 4 Fürst-Stirum-Klinik Bruchsal, Gutleutstraße 1-14, 76646 Bruchsal, Germany

**Keywords:** Breast Cancer, Cancer early detection, Sub-Saharan Africa, Health care seeking behavior, Health care system

## Abstract

**Background:**

The prevalence of breast cancer has been increasing in sub-Saharan Africa over the last few years. Patients often present with late stage disease, resulting in a high mortality rate. This study aims to estimate the prevalence of breast -related symptoms in the female population of Burkina Faso. The findings can be used to advise on adequate diagnostic health services for breast symptoms to ensure early detection and down-staging of breast cancer.

**Methods:**

A cross-sectional, population based study of 996 women was conducted to investigate the proportion of women with breast-related symptoms. A semi-structured questionnaire was used to collect sociodemographic data, information about breast cancer knowledge and details about breast-related symptoms, health care seeking and medical care. Breast-related symptoms were categorised as currently present/not currently present to estimate the current prevalence of women requiring a diagnostic service.

**Results:**

Among the 996 women, 120 reported having had a breast-related symptom in their life. Only 36 women sought medical advice and eight women had diagnostic confirmation by histological or imaging techniques. Current breast-related symptoms were reported by 33 women (3.3% of the sample). An extrapolation to Burkina Faso’s female population suggests that 184,562 women are in current need of diagnostic services due to any breast-related symptoms.

**Conclusions:**

Imaging techniques at the community and referral level are needed in order to triage women with breast-related symptoms. Specialised services need to be strengthened to ensure appropriate diagnosis and treatment of breast diseases. Education campaigns among the general population and among health care professionals are required to increase awareness of breast cancer and improve prompt health care seeking and referral.

## Background

The rise of non-communicable diseases (NCD) has become an important issue for health-care systems. Over the last few decades, national and international public health agencies have focused on controlling infectious and tropical diseases in developing countries. In developing countries, 80% of deaths occur due to NCD [1], and deaths from NCDs are predicted to increase in African countries by 27% over the next 10 years [2]. In 2012, the prevalence of cancer was estimated to be higher in developing countries, with more than eight million cases, compared to more developed regions, with more than six million cases. At the same time, breast cancer was diagnosed in about 25% of all newly diagnosed cancers worldwide, and became the most frequent cause of cancer death for women in less developed regions, with 324,000 cases per year [3]. The reasons for the increasing incidence of breast cancer in developing countries include previous underdiagnoses as well as changes in lifestyle and reproductive behaviours and a higher life expectancy [4]. In 1994, one of the goals of the International Conference on Population and Development was to make prevention and treatment of breast cancer part of the reproductive system, and globally accessible by 2015 [5]. The reality is, that case fatality and survival rates [6, 7] of breast cancer still differ remarkably between developing and developed countries. While women diagnosed with breast cancer in the United States have an 85–90% chance of five-year age-standardized survival [8], women with breast cancer in Gambia, one of the lowest-income countries in Africa, have only a 12% chance of 5-year age-standardized survival [9].

It is expected that, by 2025, the maternal mortality rate in developing countries will continue to decrease and breast cancer mortality will increase, so that both factors will be nearly identical among women of reproductive age [10]. This implies an immense challenge for resource-poor countries in which facilities for diagnostics and therapy are very limited, and the cost of suitable breast cancer care generally outweighs the monthly income [11]. Several studies from sub-Saharan Africa have shown that the majority of breast cancer patients are diagnosed at a late stage, making treatment more difficult and less effective [12, 13]. Factors that explain late presentation to health care providers have been described at the individual level (low breast cancer awareness and knowledge, misconceptions and mythical beliefs, mistrust in the health care system, financial and access barriers) and at the health system level (low quality and availability of health care services, absence of specialised public services and necessary drugs, high diagnosis and treatment costs, low knowledge and no referral among health professionals, lack of health insurance) [14–18]. Considering these difficulties, the problem may be tackled at its roots by implementing intensified research regarding risk factors for breast cancer, focusing on the creation of awareness and on early detection programs to prevent deaths from breast cancer. Breast cancer screening programs are implemented nationwide in high-income countries, but are lacking and likely to be ineffective and unaffordable in Low- and Middle Income Countries (LMICs) with a young population and insufficient local treatment options [19].

Early detection of breast cancer requires verification of all breast symptoms. Raising knowledge and awareness about breast cancer in a population requires a properly working health system where adequate breast health care is ensured for every woman who notices breast-related symptoms. To guide the development and strengthening of such a health care system, it is important to estimate the prevalence of breast symptoms which need diagnostic service, and likely follow-up and treatment. Burkina Faso, according to the Human Development Index, is one of the five least developed countries in the world. Health care is very limited and no general health insurance coverage is available. It is estimated that 1144 women develop breast cancer every year in Burkina Faso, and every second woman will die from it [3]. In the capital’s university medical centre, the main tertiary referral centre of Burkina, there is only one breast cancer specialist and very limited diagnostic and therapeutic options. Radiotherapy is non-existent throughout the whole country, despite the fact that it is estimated there could be a benefit, in terms of survival, for 50–60% of cancer cases [20].

This study aimed to estimate the prevalence of breast-related symptoms, breast cancer knowledge, health care seeking behaviour and diagnostic needs among women in Burkina Faso.

## Methods

### Setting

A cross-sectional population-based survey was conducted to assess the diagnostic needs and barriers to health care seeking with a semi-structured questionnaire among females in Burkina Faso. Burkina Faso’s population is divided into counting units, the “Zones de dénombrement”. There are 1217 urban and 11,165 rural zones in which each zone has about 1000 habitants [21]. After stratification by urban and rural population five districts (Secteur 15, Secteur 20, Secteur 23 of the capital for urban areas and the villages Boena and Siralo for rural areas) were randomly selected, proportional to population size.

The northern areas of Burkina Faso were excluded due to political disturbances.

### Data collection

The data collection took place from mid-December 2015 until the end of February 2016. According to the results of a previous study [6], which investigated the prevalence of breast masses in Ruanda and Sierra Leone as identified by the Surgeons Overseas Assessment of Surgical Need (SOSAS), a sample size of 945 women was calculated based on the assumption of 4% prevalence and the required precision of +/− 1.25% for the 95% confidence interval. Women were eligible for inclusion if they were 18 years of age or older and if they gave voluntary informed consent for participation. A total of 996 women were interviewed. Local medical students were trained by one of the investigators in terms of breast health, sampling strategies and interview techniques. Data collection was conducted via face to face interviews. In each selected district, a pencil was thrown in the air at a public place, and the direction in which the pencil tip pointed indicated the starting point for the interviews. Consecutively, in each household, one female member of each family, aged 18 years or older, was interviewed until the number of women to be surveyed in the selected district was reached. All women except ten agreed to participate in the study.

The questionnaire was composed in French, but individually translated to the local language. The questionnaire included parts of the SOSAS survey [22], in order to obtain information about the lifetime incidence and current prevalence of breast-related symptoms, as well as diagnostic need and barriers to health care seeking. The questionnaire was piloted among 15 women. Understanding of the questions was good and only minor changes were made to improve fluency of the interviews. The questionnaire was subdivided into five different chapters: sociodemographic and socio-economic situation (A), risk factors for breast cancer (B), past or present breast pathologies (C), knowledge about breast cancer and breast-self-examination (BSE) (D) and barriers to seeking health care (E). After the interview, each woman received training on breast symptom awareness and self-examination. In the case of current suspicious self-reported symptoms, referral to a health care centre was ensured.

During the interview, answers were noted on the questionnaire and afterwards were checked for consistency and entered in a database (MS ACCESS 2016) by one of the investigators.

### Statistical analysis

The main question was about presence or ever presence of breast-related symptoms. Generally we assumed all breast-symptoms would need diagnostic assessment to rule out breast cancer. For descriptive purposes, leading breast-related symptoms were classified into groups (e.g. painful tension, breast mass) and whether they were associated with breastfeeding or not. The current status of women who reported any breast-related symptoms in their lifetime was categorised as follows: women who had a diagnostic service, women who had a surgical intervention without any diagnosis, women who had no current symptoms and women who currently had unverified symptoms. The percentage of currently unverified symptoms was used to extrapolate the results to Burkina Faso’s female population. Regarding the age structure, our study sample is representative for the general population of Burkina Faso, as age groups within the study sample are comparable to the ones listed in the World Fact Book of 2016 [23]. Descriptive data analysis was performed using SPSS 19.

## Results

Data from 996 interviewed Burkinabe women, aged 18 years or older, was analysed. Sociodemographic data is described in Table 1.

**Table 1:** Sample characteristics, all women (*n* = 996)

	n %
Age in years [m], (sd)	33.4 (12.5)
Age group	
18–30 years	529 (53.1)
31–45 years	302(30.3)
46–86 years	158 (15.9)
Missing	7 (0.7)
Highest level of education	
None	557 (55.9)
Elementary school	192 (19.3)
Secondary school	134 (13.5)
Tertiary school	57 (5.7)
University	55 (5.5)
Missing	1 (0.1)
Literate	
Yes	400 (40.2)
No	596 (59.8)
Occupation	
Housewife	167 (16.8)
Farmer	396 (39.8)
Business	323 (32.4)
Public service	25 (2.5)
Student	47 (4.7)
Other	38 (3.8)
Married	
Yes	795 (79.8)
No	201 (20.2)
Religion	
None	2 (0.2)
Islam	721 (72.4)
Christian	271 (27.2)
Missing	2 (0.2)
Residence	
Urban	583 (58.5)
Rural	413 (41.5)
Births	
0	157 (15.7)
1–3	459 (46.0)
3+	380 (38.2)
Nursing period	
No children	157 (15.7)
< 12 months	6 (0.6)
12–24 months	402 (40.4)
24–36 months	404 (40.6)
> 36 months	12 (1.2)
Missing	15 (1.5)

### Breast cancer knowledge

**Fig. 1:**
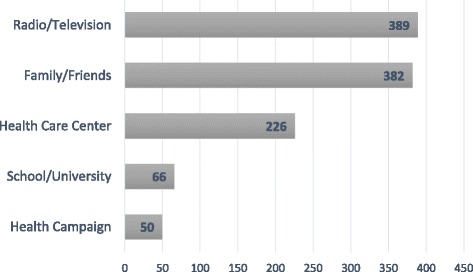
Sources of information about breast cancer, multiple answers were possible (*n* = 749)

Women were asked to rate their subjective knowledge about breast cancer on a scale between 1 (good) and 10 (bad). The average score was 8,29. This low self-rated level of knowledge was also evident in other breast cancer knowledge-related questions. A single open question concerning risk factors for breast cancer was answered by 87% of all women as ‘no idea’. A minority stated nutrition (*n* = 57), reproductive reasons (*n* = 19), heredity or age (*n* = 7) as “correct” risk factors. Other factors stated were mechanical reasons like carrying money or cell phones under the bra (*n* = 60), use of aesthetic products (*n* = 15), and infection or externals (*n* = 14). A minority of all women (11.3%) stated that they knew about BSE. Questions concerning the time-point and frequency of practicing BSE were answered correctly by only 14 and 26 women, respectively. One quarter of all women had never heard about breast cancer. Among those who had heard about it, almost 25% were not sure if breast cancer could be deadly if not treated. The 749 women who had heard about breast cancer were asked where they had heard about it. Results are shown in Fig. 1.

### Breast related symptoms

Having had breast-related symptoms at least once in their lifetime was reported by 120 women. The reported primary symptoms are described in Table 2. In 65% of all cases, the symptoms were reported as associated in time with breastfeeding**.** Out of the 120 women with breast-related symptoms, 30% presented to a health care provider to get medical advice (Table 2). The reasons for not seeking medical advice were primarily absence of disability associated with the symptoms, which led to symptom ignorance, as well as lack of money, no confidence in doctors, no access to health care centres and shame about the disease. A diagnostic confirmation by histopathology or imaging techniques was reported by eight women, and five women reported having had surgical treatment without any diagnostic confirmation (Table 2). Among 74 women who reported having had breast symptoms in the past but had no current symptoms, 28 received medical treatment from a health care facility, 32 used a form of self-medication or went to a traditional healer and in 14 cases, symptoms were self-limiting. Current unverified symptoms were reported by 33 women (3.3% of the study sample). In 16 cases, the symptoms were associated with breastfeeding. Taking into account that Burkina Faso’s female population aged over 15 years is estimated to be 5,592,800 women, it can be estimated that 184,562 women would be in current need of diagnostic services due to any breast-related symptoms.

**Table 2:** Primary symptoms, health seeking and current status, all women with breast-related symptoms (*n* = 120)

	n %
Primary symptoms	
Painful tension	33 (27.5)
Painful tension with appearance of purulence	25 (20.8)
Pain	17 (14.2)
Breast mass	23 (19.2)
Painful tension with redness	12 (10.0)
Changing of the nipple	4 (3.3)
Itchiness	4 (3.3)
Redness	2 (1.7)
Health seeking after first symptoms	
Medical advice	36 (30.0)
Traditional medicine	32 (26.7)
Nothing	52 (43.3)
Current status	
Had diagnostic service	8 (6.7)
Surgery without diagnostic service	5 (4.2)
No current symptom	74 (61.7)
Current symptom	33 (27.5)

## Discussion

In our study population, 12% of all interviewed women reported having had any breast-related symptoms at least once in their lifetime, and 3.3% had a current unverified breast-related symptom at time of the interview. The extrapolation to the female population of Burkina Faso shows a current need for diagnostic service among 184,562 women in Burkina Faso. Symptoms are rarely related to breast cancer, but a medical check-up with a minimum of ultrasound diagnosis would ideally be recommended to every one of these women. Generally, a sufficient diagnostic service concerning breast symptoms would include availability of physician’s CBE (Clinical Breast Examination), ultrasound, biopsies or fine-needle aspiration, and mammography in specialised centre.

In fact, there are little or no facilities for breast health care, and structural barriers exist in Burkina Faso’s hierarchically organized national health care system. In the case of breast-related symptoms, women would typically first visit the broadly available very basically equipped reference centres, called CSPS (Centre de Santé et de Promotion Sociale, 1760 centres). No doctors and no surgery facilities are available at these centres; similarly, the next highest reference step, called CMA (Centre Médical avec Antenne Chirurgicale, 47 centres), also does not provide infrastructure for breast care. According to the newest guidelines of the BC2.5 initiative at the Fred Hutchinson Cancer Research Centre [24], training regarding CBE among health care providers in the very basic health care reference levels, or among trained non-physician providers, education about risk factors and symptoms, and creation of awareness of breast cancer among both medical staff and the general population, may improve survival, lower morbidity and reduce the cost of care. A study in Sudan suggested that in breast-examination trained laywomen can provide breast cancer screening [25], and a Tanzanian study reported a down staging of breast cancer in a program to downstage cancer by trained lay personnel [26]. It should be noted that medical specialists are available at the third level reference, called CHR (Centre Hospitalier Regional, 11 centres) and CHU (Centre Hospitalier Universitaire, 5 centres), but these too are not sufficiently equipped with breast ultrasound or diagnostic mammography for breast care.

In our study, only 30% of 120 women who reported having had any breast-related symptoms in their life sought medical advice. Common reasons for not seeking care were ignorance and lack of money; this is consistent with other studies which have reported these factors to be barriers to health care seeking among breast cancer patients in sub-Saharan Africa [6, 17, 27]. Another reason for not seeking health care might be the low level of knowledge about breast cancer, which was evident in our study and was also reported in several other studies from Sub-Saharan Africa, mainly among breast cancer patients [6, 14, 15, 17]. Furthermore, the mentioned risk factors (e.g., carrying money or mobile phones in the bra) for breast cancer reflect notions of stigma and myths which may come along with breast-related symptoms; these might hinder women to seek health care, but might also be related to the use of traditional medicine, as was reported in the neighbouring countries of Ghana [28] and Mali [17].

To improve the situation of breast health care in Burkina Faso, in accordance with the above-mentioned guidelines [24], we suggest that it is imperative to increase awareness and knowledge about breast cancer among the general female population. Awareness and education campaigns have to consider the low literacy rate among women in Burkina Faso, and might be best suited as part of maternal health care services. Teaching of BSE could additionally create awareness and early symptom detection.

However, without adequate diagnostic and treatment facilities, awareness campaigns alone cannot lead to improved breast cancer care and survival. Therefore, ultrasound facilities need to be allocated to every CMA, which means at least 47 instruments for the whole country. Breast ultrasound is helpful to distinguish harmless fluid-filled cysts from suspect solid masses, and can be helpful to provide additional information about the extent of the tumour [29], especially against the background that a high number of breast cancer patients in LMICs present with locally advanced breast cancer. Moreover, mammography is currently the most effective screening tool for breast cancer. This is recommended for women aged 50 years and older, but not for the younger population [19]. In terms of diagnostic mammography, five mammograms in total are needed to ensure adequate equipment in each CHU, and specialists in analysing both ultrasound and mammography imagery need to be provided. The Burkinabe government has to put more effort in to upgrading pathology services, which are still limited to the CHU and private clinics. Establishing infrastructure for telepathology and assisting pathology laboratories might be a solution.

To strengthen the health system as proposed, support from international stakeholders is needed, as well as public private partnerships. To ensure that the standard treatment options for breast cancer, such surgery, radiation therapy and systemic therapy, are accessible to all patients in LMICs such as Burkina Faso, universal health coverage might be a solution in the long run.

Our study gives a representative picture of breast-related symptoms in the Burkinabe female population. There are some limitations that should be noted. First, the breast symptoms were self-reported and there may have been recall bias, as minor symptoms that appeared a long time ago might have been missed. Therefore, we assume that actual figures regarding lifetime incidence of breast-related symptoms are probably much higher than reported. Secondly, we did not perform an actual on-site assessment of the health facilities; for logistic reasons, we only obtained information about public diagnostic facilities from local health professionals. Health facilities may possibly have better equipment than the overall lack which we report here, especially as private clinics were not considered.

## Conclusions

Only 6,7% of all women with breast symptoms had received a diagnostic confirmation by histopathology or imaging techniques and 4,2% reported having had surgical treatment without any diagnostic confirmation. There is a huge need to improve diagnostic service and its utilisation for breast symptoms in Burkina Faso’s health care system.

An extrapolation showed a current need for diagnostic services among an estimated 184,562 women with breast-related symptoms in Burkina Faso. Awareness on need for diagnostic assessment of breast symptoms among the general female population has to be increased, and solutions to improve health worker’s education and to strengthen the health care system, including upgrading of diagnostic and treatment facilities, are needed.

## Abbreviations

BSE: Breast-Self-Examination
CBE: Clinical Breast Examination
CHR: Centre Hospitalier Regional
CHU: Centre Hospitalier Universitaire
CMA: Centre Médical avec Antenne Chirurgicale
CSPS: Centre de Santé et de Promotion Sociale
LMICs: Low- and Middle-Income Countries
NCD: Non-Communicable Diseases
SOSAS: Surgeons Overseas Assessment of Surgical Need
